# A knowledge base for the discovery of function, diagnostic potential and drug effects on cellular and extracellular miRNAs

**DOI:** 10.1186/1471-2164-15-S3-S4

**Published:** 2014-05-06

**Authors:** Francesco Russo, Sebastiano Di Bella, Vincenzo Bonnici, Alessandro Laganà, Giuseppe Rainaldi, Marco Pellegrini, Alfredo Pulvirenti, Rosalba Giugno, Alfredo Ferro

**Affiliations:** 1 Laboratory of Integrative System Medicine, Institute of Informatics and Telematics and Institute of Clinical Physiology, National Research Council, Pisa, Italy; 2 Department of Computer Science - University of Verona, Italy; 3 Department of Molecular Virology, Immunology and Medical Genetics Comprehensive Cancer Center - The Ohio State University, OH; 4 Department of Clinical and Molecular Biomedicine - University of Catania, Italy

## Abstract

**Background:**

MicroRNAs (miRNAs) are small noncoding RNAs that play an important role in the regulation of various biological processes through their interaction with cellular mRNAs. A significant amount of miRNAs has been found in extracellular human body fluids (e.g. plasma and serum) and some circulating miRNAs in the blood have been successfully revealed as biomarkers for diseases including cardiovascular diseases and cancer. Released miRNAs do not necessarily reflect the abundance of miRNAs in the cell of origin. It is claimed that release of miRNAs from cells into blood and ductal fluids is selective and that the selection of released miRNAs may correlate with malignancy. Moreover, miRNAs play a significant role in pharmacogenomics by down-regulating genes that are important for drug function. In particular, the use of drugs should be taken into consideration while analyzing plasma miRNA levels as drug treatment. This may impair their employment as biomarkers.

**Description:**

We enriched our manually curated extracellular/circulating microRNAs database, miRandola, by providing (i) a systematic comparison of expression profiles of cellular and extracellular miRNAs, (ii) a miRNA targets enrichment analysis procedure, (iii) information on drugs and their effect on miRNA expression, obtained by applying a natural language processing algorithm to abstracts obtained from PubMed.

**Conclusions:**

This allows users to improve the knowledge about the function, diagnostic potential, and the drug effects on cellular and circulating miRNAs.

## Background

MicroRNAs (miRNAs) are small (~22-nucleotide) non-coding RNA molecules that are single-stranded in their functional form and act as post-transcriptional regulators of gene expression [1]. Their importance was confirmed in several cellular processes like development, proliferation, and apoptosis. Moreover, altered miRNA expression profiles have been linked to a large number of pathological conditions, such as cancer, suggesting that miRNAs are involved in disordered cellular function. miRNA expression profiles have been shown as potential signatures for the classification, diagnosis, and progression of cancer [2,3]. Recently, a significant amount of miRNAs has been found in extracellular human body fluids [4,5]. Some circulating miRNAs in the blood have been successfully revealed as biomarkers for several diseases including cardiovascular diseases, [6] and cancer [4,7].

miRandola is the first comprehensive database of extracellular circulating miRNAs [8]. The database represents a useful reference tool for anyone investigating the role of extracellular miRNAs as non-invasive biomarkers as well as their physiological function and their involvement in diseases. miRNAs are classified into different categories, based on their main extracellular forms: complexed with Argonaute2 (Ago2) proteins [9,10], encapsulated within exosomes [11] or bound to high-density lipoprotein (HDL) [12]. Exosomes are 50-nm to 90-nm vesicles arising from multivesicular bodies and released by exocytosis [13]. They consist of a limiting lipid bilayer, transmembrane proteins and a hydrophilic core containing proteins, mRNAs and miRNAs. Exosomes may horizontally transfer RNAs, including miRNAs that have been shown to be functional after exosome mediated delivery [11]. It has been reported that a significant portion of circulating miRNAs in human plasma and serum is associated with Ago2 [9,10]. Ago2 is the effector component of the miRNA-induced silencing complex (miRISC) that directly binds miRNAs and mediates messenger RNA repression in cells [14,15]. The high-density lipoprotein (HDL) is a delivery vehicle for the return of excess cellular cholesterol to the liver for excretion. Recently, it has been reported that HDL transports endogenous miRNAs and delivers them to recipient cells with functional targeting capabilities [12] providing evidence that HDL-miRNAs could potentially serve as novel diagnostic markers in much the same way exosomal miRNAs have.

This is now emerging as a hot, quickly developing research topic due to the promising role of extracellular miRNAs as non-invasive biomarkers. miRandola constitutes a useful environment for the study of reported circulating miRNAs and may help prioritize their systematic clinical evaluation. Here we present some new tools that we developed and incorporated into miRandola to help the discovery of function, diagnostic potential and drug effects on cellular and extracellular miRNAs in order to facilitate user investigation of circulating miRNAs.

## Content and utility

### A database for extracellular miRNAs

miRandola is a comprehensive database of extracellular circulating miRNAs. It provides a variety of information including associated diseases, samples, methods used to isolate miRNAs, the description of the experimental protocol and the potential biomarker role.

Data is collected from ExoCarta [16], a database of exosomal proteins, RNA and lipids and PubMed (*www.ncbi.nlm.nih.gov/pubmed/*). The database is manually curated and constantly updated by the authors and the scientific community who can give its contribution to the project by submitting new data about extracellular miRNAs. The current version of the database contains 119 papers, 2276 entries, 590 unique mature miRNAs and 23 types of samples.

miRNAs are classified into four categories, based on their extracellular form: miRNA-Ago2 (173 entries), miRNA-exosome (856 entries), miRNA-HDL (20 entries) and miRNA-circulating (1227 entries). The latter is used when information about the specific miRNA carrier is not available and constitutes the largest group.

The database is implemented in MySQL running on an Apache server and it is equipped with a PHP web interface. Users may query the database by mature miRNA, miRNA family, sample, diseases, malignant cell lines, and potential biomarker role, to get information about the diseases, processes and functions in which the corresponding miRNAs are involved and the tissues in which they are expressed. Results consist of published data about the searched items and predictions computed by miRo', a web knowledge base which contains miRNA functional annotation inferred through their validated and predicted targets.

### A systematic comparison of expression profiles of cellular and extracellular miRNAs

**Figure 1:**
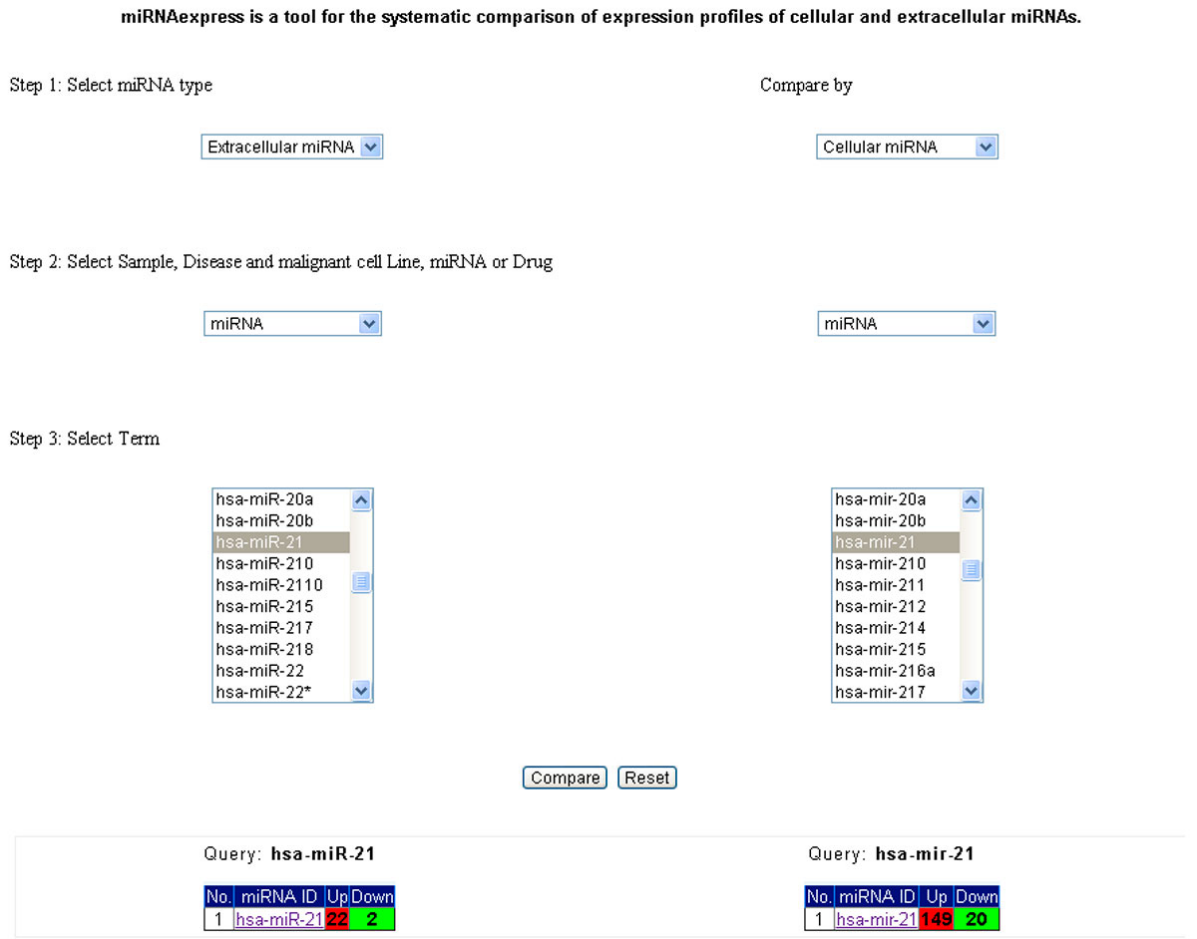
**miRNAexpress web interface**. In the step one users select the miRNA type (cellular or extracellular); in the step two they choose the sample, disease and cell line, miRNA or drug; in the step three users specify the terms of interest (such as hsa-miR-21). After clicking on the Compare button users will see the number of up and down regulated miRNAs.

We extended miRandola with miRNAexpress, the first tool for the systematic comparison of expression profiles of cellular and extracellular miRNAs. In three simple steps (Figure 1) users can compare 1) cellular-cellular miRNA expression, 2) cellular-extracellular miRNA expression or 3) extracellular-extracellular miRNA expression. First, the user must specify the miRNA form between cellular and extracellular, followed by the category of objects to compare among sample, disease and cell lines, miRNA and drugs. Finally, specific instances of the objects to compare must be selected, e.g. 'plasma' and 'cancer'. The system shows the results of the comparison as two lists of up- and down-regulated miRNAs related to the selected objects.

**Figure 2:**
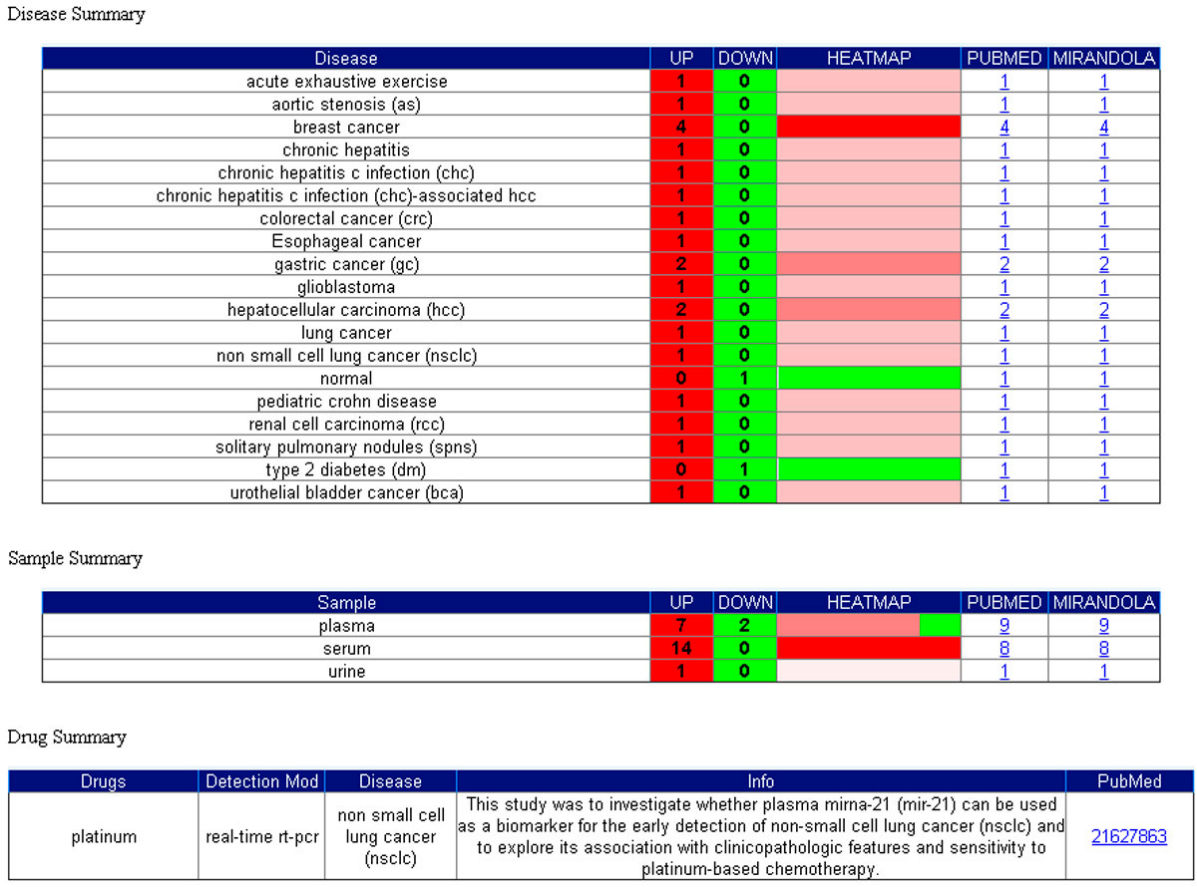
**miRNAexpress details section**. The details section shows the Disease, Sample and Drug summaries for each selected miRNA with the number of up and down regulated miRNAs, the heatmap, links to PubMed, Phenomir and miRandola.

Cellular miRNA data is taken from PhenomiR [17], while extracellular miRNA information is obtained from the miRandola database. The details section (Figure 2) shows the Disease, Sample and Drug summaries for the selected miRNA. It contains information on the up- and down-regulation, the heatmap, links to PubMed, Phenomir and miRandola. Moreover it shows a histogram of miRNA expression in diseases with up and down regulation. Through miRNAexpress users can observe the expression profile pattern for each miRNA in a specific sample or condition. In Figure 1 we report an example of analysis for hsa-miR-21. In this case, the expression profile patterns for the cellular and extracellular forms are the same (upregulation).

In order to infer the effect of drugs on miRNA expression we downloaded the lists of all miRNAs and drug names from miRBase [18] and drugbank [19], respectively. For each miRNA, we queried PubMed through the Entrez utilities [20] in order to retrieve all the papers whose titles and abstracts contained the name of the miRNA. We selected the abstracts that contained the name of the miRNA and the name of a drug. If such miRNA and drug co-occured in the same sentence, this sentence was stored as support of the relation miRNA-Drug. Sentences were extracted from the text by using the Stanford NLP software [21,22]. We parsed each sentence in order to find triples containing subject, verb and object, where subject and object were either miRNA or drug names and the verb indicated the effect of the relationship miRNA-Drug. We performed this task by using the software ReVerb [23,24]. Next, we manually parsed the selected data in order to keep only those relations concerning drugs that are known to up or down regulate a miRNA, together with the related disease and the used experimental platform (microarray, Northern blot, etc.). We enriched our knowledge base by adding supportive information taken from SM2mir [25], a manually curated databases which maintains experimentally validated effects of small molecules on miRNA expressions (last update refers to June 2012). The final results thus obtained from the above pipeline are presented in the Drug summary and consist of the PubMed ID of the paper, the support sentence and, if specified, the related disease and the experimental techniques.

### miRNA targets enrichment analysis

DAVID [26] is a system for gene functional annotation and enrichment. We now extended miRandola with miRto, a tool which integrates DAVID functional annotation (through the available web service module [27]) with target prediction (TargetScan *www.targetscan.org/vert_*61*/ *and miRanda *www.microrna.org/microrna/home.do*) and validated targets (miRTarbase *mirtarbase.mbc.nctu.edu.tw/*) tools.

**Figure 3:**
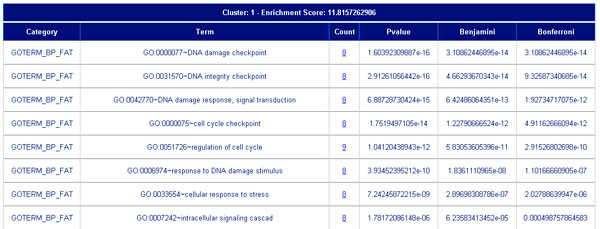
**The functional annotation clustering showed using miRto**. The functional annotation clustering integrates kappa statistics to measure the degree of the common genes between two annotations, and fuzzy heuristic clustering to classify the groups of similar annotations according kappa values.

**Figure 4:**
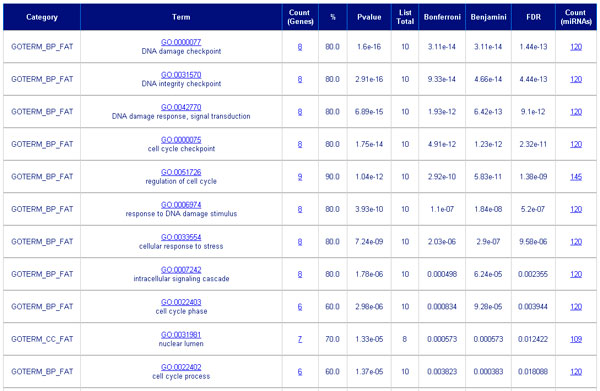
**The functional annotation chart showed using miRto**. The functional annotation chart is an annotation-term-focused view which lists annotation terms and their associated genes under study. We report in the chart the number of miRNAs that bind targets in the user list. After clicking on the count, users will see the target prediction for both cellular and extracellular miRNAs.

In the first step users can paste a list of miRNA targets and in the second step users can set filters for Gene Ontology terms, KEGG Pathways, number of targets and p-value. After clicking on the search button two analyses will be available. The Functional Annotation Clustering (Figure 3) is based on the hypothesis that similar annotations should share gene members. It integrates a variant of the Kappa statistics to measure the degree of common genes between two annotations, and a fuzzy heuristic clustering to classify the groups of similar annotations according to kappa values. Accordingly, the more common genes annotations share, the higher chance they will be grouped together. The p-values associated with each annotation term inside each cluster are based on Fisher Exact/EASE Score [28]. The Group Enrichment Score is used to rank the annotations' biological significance. Thus, the top ranked annotation groups most likely have consistent lower p-values for their annotation members. The Functional Annotation Chart (Figure 4) is an annotation-term-focused view which lists annotation terms and their associated genes under study. We report in the chart the number of miRNAs that bind targets in the specified user list. After clicking on the count, the system will show the target prediction for both cellular and extracellular miRNAs.

## Discussion

**Figure 5:**
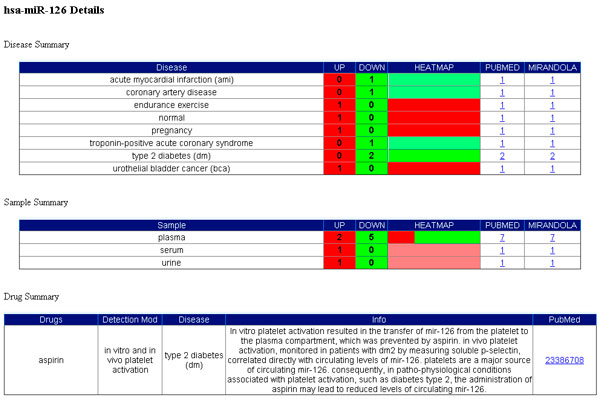
**Drug effects on miRNA expression. Results obtained by using miRNAexpress web interface**. In miRNAexpress users can select miR-126 and discover that aspirin produces the down-regulation of the miRNA in patients with type 2 diabetes, thus the use of platelet inhibitors affect the plasma levels of miR-126.

Circulating miRNAs appear to be affected by various parameters, including drugs. For instance, De Boer and colleagues [29] have shown that aspirin intake should be accounted for when considering circulating miR-126 as diagnostic biomarker for cardiovascular diseases, or, more generally, when studying the possible role of miRNAs as mediators of cardiovascular disease. Using miRNAexpress users can select miR-126 (the specific miRNA of interest) and discover that aspirin (the related drug) produces the down-regulation of the miRNA in patients with type 2 diabetes, thus the use of platelet inhibitors affect the plasma levels of miR-126 [29]. This result is showed in Figure 5.

Drugs can also determine the up-regulation of extracellular miRNAs. For instance, by selecting acetaminophen in miRNAexpress users will see that this drug up-regulates miR-122 and miR-192 in acetaminophen-induced acute liver injury (APAP-ALI) providing the evidence for the potential use of miRNAs as biomarkers of human drug-induced liver injury [30]. One apparent premise to using extracellular miRNAs for disease diagnose is the notion that the abundance of the miRNAs in body fluids reflects their abundance in the malignant cells causing the disease, thus researchers have focused on miRNAs that are abundant in the cells of origin.

Using miRNAexpress users can systematically compare cellular and extracellular expression profiles for a specific disease, and find instead that extracellular miRNAs do not necessarily reflect the abundance of miRNAs in the cell, as demonstrated by recent studies [31]. For instance, by selecting prostate cancer disease (in step 3 of the tool), for both extracellular and cellular miRNAs, users will see that there is no difference in the expression profile patterns for some miRNAs (e.g. let-7c, let-7e and miR-107), while there are some differences for other miRNA signatures (e.g. miR-141 is up-regulated in the plasma of prostate cancer patients [32], and is down-regulated in prostate cancer cell lines [33]).

## Conclusions

We presented useful tools for further understanding the role of cellular and extracellular miRNAs in the context of their targets, regulation and drug effects on their expressions and annotations. These tools extended the miRandola database, the only online resource collecting information about extracellular miRNAs.

## Authors' contributions

FR and RG conceived of the study and organized the manuscript; SDB and VB participated in the design of the study; FR, SDB and VB carried out the implementation. FR, SDB, AL, VB, RG, AP, RG, MP, and AF analyzed the data. All authors wrote, read and approved the final manuscript.

## Availability and requirements

miRandola is available at http://atlas.dmi.unict.it/mirandola/

## Competing interests

The authors declare that they have no competing interests.

## Declarations

This article is published as part of a supplement. The publication costs for this article were funded by PROGETTO BANDIERA INTEROMICS "Sviluppo di una piattaforma integrata per l'applicazione delle scienze "omiche" alla definizione dei biomarcatori e profili diagnostici, predittivi e teranostici" CUP B91J12000270001.

This article has been published as part of *BMC Genomics *Volume 15 Supplement 5, 2014: Italian Society of Bioinformatics (BITS): Annual Meeting 2013: Genomics. The full contents of the supplement are available online at http://www.biomedcentral.com/bmcgenomics/supplements/15/S3
